# Improving Hands-On Suture Opportunities for Medical Students: A Collaborative Initiative Between Medical Students and a Simulation Lab

**DOI:** 10.7759/cureus.66328

**Published:** 2024-08-06

**Authors:** Rebecca Mosaad, Jacob Nicodemo, Rakan Hamady, Adam Dubrowski

**Affiliations:** 1 Health Sciences, Ontario Tech University, Oshawa, CAN; 2 Faculty of Medicine and Health Sciences, McGill University, Montreal, CAN

**Keywords:** near peer learning, peer assisted learning, knot tying techniques, suturing techniques, simulation in medical training, medical education

## Abstract

Technical skills are an integral part of the practice of medicine. Simulation-based education (SBE) is a widely employed approach that allows students to acquire these skills prior to practicing them in the clinical setting. To discuss the state of SBE and potential avenues to improving education and medical student experiences, this editorial will explore the lived experiences of junior medical students, the observations of a research graduate student’s informal conversations, and an educational quality improvement (EQI) pilot conducted by students at a satellite medical campus.

Pre-clerkship Canadian medical students reported having limited opportunities to practice their technical skills. For some, these SBE sessions came at inopportune times in their academic journey, preventing them from maximizing their chances at real-world exposure. Having identified this as an issue, students sought ways to allow themselves and their peers to practice technical skills outside of the undergraduate medical curriculum, such as organizing peer and near-peer-led suturing events. Still, students feel these sessions are a start but do not adequately meet their needs, as access to practice materials is still restricted to the sparse events held by students, and experienced feedback is scant. To address these needs, we explore how simulation technology research and development labs can support peer-assisted learning by training students to teach technical skills and provide feedback to their peers. We also propose increasing access to simulation materials asynchronously to allow for practice when the students can benefit most.

## Editorial

Introduction

Today’s practice of medicine requires physicians to blend both extensive theoretical knowledge and specialized technical skills. For many Canadian medical students, pre-clerkship training has a greater focus on the acquisition of theoretical knowledge. Simulation-based education (SBE) is often utilized during this phase of training to allow students to acquire and practice these techniques before entering the clinical setting. To understand current practices of SBE in medical education and the perspectives of students on SBE, we conducted a pilot educational quality improvement (EQI) study with students at a satellite undergraduate medical school campus at a Canadian university.

Context

The pilot took place in the form of verbal feedback from nine attendees of an extracurricular suture event organized by pre-clerkship medical students in collaboration with a recent medical graduate. Open to all medical students attending this university, suture events, such as this one, have been run for a number of years, taking place at the satellite campus. These events are typically directed by a former student mentor, who helps guide participants in practicing suturing skills. These workshops provide students with the opportunity to gain exposure to surgery, learn basic operating room (OR) etiquette, and learn and practice the fundamentals of suturing. Instruments and space to practice were provided for by the university, while the models used were provided by maxSIMhealth, a simulation lab situated at Ontario Tech University (Oshawa, Ontario, Canada). This research lab focuses on developing simulation tools and integrating SBE into student learning.

Students at this event reported that surgical SBE integrated into their undergraduate medical curriculum is limited. In-curriculum exposure to suturing was contained in a four-hour session taught by a practicing surgeon. This session covered both the theory and practice of the technique, as well as other common OR procedures (nasogastric tube insertion, Foley catheter insertion). For many, this session occurred after having completed or partially completed their pre-clerkship two-week surgical rotation. Another approach previously used is referred to as decentralized SBE (or de-SBE), which provides users the opportunity to practice outside of a formal lab [1]. No models for decentralized are currently available to students to practice suturing. Thus, students believe they may have missed opportunities to practice skills in real OR settings, as they did not have the opportunity to first build these skills in a simulated environment.

Among the students present for our EQI study, some had previously attended surgical academic conferences. Having not previously participated in SBE at an academic conference, respondents did not view these conferences as opportunities for skills development and affirmed that they would not attend such events solely for skills training opportunities. However, they were favorable to integrating technical skills training into academic conferences.

Having animated numerous suture workshops in the past and having significant OR experience as a senior medical student, the tutor of this workshop was confident in her ability to teach basic suturing skills, knot-tying techniques, and OR etiquette. This was due to her additional experience in OR training. However, those attending the workshop expressed that most senior students and recent graduates do not have the experience and skills necessary to lead such events confidently due to limited in-course and extracurricular opportunities. Additionally, given the location of a satellite campus, surgical residents are not available to assist with these events.

Honing technical skills requires deliberate, continuous practice in the form of directed simulations, independent practice, and real-world practice [2]. As such, students have identified this dearth of exposure to technical skills as a challenge and, indeed, have attempted to address this need for themselves alongside their peers. This is accomplished through many events run on-campus by students, not limited to suture workshops such as that of our pilot EQI study. Students reported having two to three peer-coordinated suture events organized annually, generally with senior students, mainly through self-guided activities.

Current gap

From our discussion, the first gap we identified in the SBE of technical skills is the lack of exposure as perceived by the students. Students report that opportunities for technical practice in the curriculum are sparse and that simulation materials are difficult to access outside of the infrequent extracurricular suture events. This limited practice time resulted in students missing opportunities to use their skills in a real OR setting.

The second gap we identified was the lack of assessment from an experienced teacher during extracurricular self-guided suture events. Participants of our pilot benefitted from the presence of a motivated tutor with significant OR experience throughout their education. However, the tutor expressed that in her experience, although senior students may have completed their core surgery rotations, many do not feel prepared to teach technical skills and lead SBE events.

Involving the students in the solution

Equipping the Students

Simulation research and development labs can play a vital role in supporting student-led initiatives aimed at increasing the accessibility to SBE training materials. Such labs have developed affordable and reusable surgical education models aimed at teaching technical skills. These models allow students to practice skills of various difficulty levels. For instance, suture pads allow students to build their confidence in this basic skill, with the goal of ultimately translating it to the clinical setting. More advanced simulators, such as those for bowel anastomosis, provide students with additional exposure to the field of surgery, fostering their interest in a career in surgery. Design files are often made public to enable students with access to 3D printers to print their own simulators or molds for silicone simulators that they can then recreate on their own.

In many instances, such as the Canadian Conference for the Advancement of Surgical Education (C-CASE), students had previously expressed a need for more technical skills practice opportunities. Thus, organizers introduced a series of five workshops to the 2023 iteration in collaboration with the simulation experts from maxSIMhealth using a variety of models. Surgical education conferences, such as C-CASE, offer a unique opportunity to bring motivated students and simulation labs in touch. It is encouraged to continue running and developing conferences with this model to maximize participant experience.

Training Students to Train Students

Cliché as it may be, there is great merit to the old proverb, “see one, do one, teach one.” The thoughtful implementation of this axiom among medical students interested in surgical education guides our second proposal, the creation of resources and training programs to support peer and near-peer-assisted learning programs (P/NPAL).

As defined by Alkhail, “peer-assisted learning is an umbrella term encompassing a variety of collaborative and cooperative educational strategies, including peer teaching and learning, peer assessment, peer mentoring, and peer leadership” [3]. Effective near-peer teaching can be implemented by equipping senior medical students with the knowledge and skills to lead SBE workshops and provide appropriate feedback to participants. A secondary positive outcome to this approach is that “pairing junior and senior undergraduate students establish(es) psychological and social support system(s) and aids professional and personal development” [3]. The role of the tutor also allows senior medical students to grow their competencies in CanMEDS roles, such as that of the leader, communicator, and collaborator.

Such P/NPAL models are effective at increasing participant efficacy and confidence. A P/NPAL flipped classroom model comprising a series of five workshops led by senior medical students and a surgical resident has been shown to significantly increase students' self-perceived confidence performing a variety of surgical knots. Participants reported they would be more likely to perform such an act in a clinical setting should the opportunity present itself [4].

Asynchronous Access to Materials

Finally, access to training material for independent practice can be facilitated by increasing access to low-cost simulation materials and online training. In addition to the development and distribution of affordable simulators, universities can allow students greater exposure to SBE by providing access to models during frequent “office-hours” style sessions, where students are free to practice as needed.

Following a flipped classroom model, students attending C-CASE (in 2024) will now have access to a pre-learning Google Classroom, which will be completed prior to attending the skills workshop at the conference. A range of materials will be provided to students, allowing them to prepare at their own pace. This mandatory preparation ensures they arrive at the conference equipped with an understanding of when to apply procedures and the correct techniques to use, allowing them to get the most out of the SBE session. This model is easily translatable to student-led initiatives.

Limitations to the study

The main limitation of the work described in this editorial is that this was not a formal research study but rather an EQI pilot. The sample size was small, and the issues identified may be specific to the satellite medical school and may not directly translate to larger programs with a bigger student body. The selection bias of those involved in the EQI is also likely, as all participants were already attending the event with the intention of pursuing professional development in this area. These skills may not be applicable to all medical specialties, and thus, the solution may not be transferable to other areas of training. A more inquisitive study might yield different results.

Future efforts and directions

Our EQI took place at a small satellite campus. Therefore, we recommend performing a thorough needs assessment at larger Canadian campuses and other faculties to determine whether similar issues are felt by students across Canada. Once needs are elaborated, an interdisciplinary team of simulation experts, curriculum developers, and medical students can be established to address the gaps in exposure to SBE in surgical education at the undergraduate medical level.

In the long term, our goal is to establish an asynchronous surgical education learning management system in collaboration with student interest groups, incorporating simulation technology research and development labs at its core. Depending on the results of the needs assessment, a de-SBE program can be envisioned integrating the three strategies proposed to address the issues identified in this pilot study:

1. Support access to instruments and simulator models

2. Train students to be effective tutors

3. Provide access to validated asynchronous learning materials

We anticipate that the models become an adjunct to in-curriculum training for students motivated to build their technical skills. Collaboration between students and simulation labs would allow for the constant collection of feedback as the program is disseminated to multiple universities to ensure the program is adjusted to the specific needs of different student populations.

We also propose the establishment of an inter-school forum. This would allow students to participate in knowledge transfers on how others across the nation are approaching education and how they can adapt their training. Most importantly, this would empower medical students to play an active role in their education, encouraging autonomy and problem-solving skills and contributing to the greater scientific community.

Conclusion

Canadian undergraduate medical students participating in these conversations feel that they do not have enough SBE opportunities to practice surgical skills prior to core clerkship rotations. Senior medical students have sought to fill in this gap by organizing extra-curriculum suture and surgical skills workshops. Nonetheless, the lack of tutors trained in P/NPAL methods and access to simulation labs and their materials is a challenge.

We believe that training senior students as tutors will help them create safe and effective learning environments. To increase access to various models, we propose the collaboration of student initiatives with simulation technology research and development labs. This will facilitate the implementation of P/NPAL extracurricular programs aimed at increased confidence in surgical skills, as well as support the development of asynchronous materials. Further investigations are encouraged to assess the impact of these interventions and to determine their generalizability to the various medical programs.
